# Reliability at the Lower Limits of HIV-1 RNA Quantification in Clinical Samples: A Comparison of RT-PCR versus bDNA Assays

**DOI:** 10.1371/journal.pone.0006008

**Published:** 2009-06-23

**Authors:** Ronald J. Lubelchek, Blake Max, Caroline J. Sandusky, Bala Hota, David E. Barker

**Affiliations:** 1 Division of Infectious Diseases, John H. Stroger, Jr. Hospital of Cook County, Chicago, Illinois, United States of America; 2 Ruth M. Rothstein CORE Center, Chicago, Illinois, United States of America; 3 Department of Internal Medicine, Rush University Medical Center, Chicago, Illinois, United States of America; Comprehensive AIDS Reseach Center, China

## Abstract

**Introduction:**

To explore whether an assay change was responsible for an increasing proportion of patients with undetectable HIV viral loads at our urban HIV clinic, we selected highly stable patients, examining their viral loads before and after changing assays. We compared the proportion with detectable viremia during RT-PCR vs. bDNA periods.

**Methodology/Principal Findings:**

We selected patients with ≥1 viral loads assessed during both RT-PCR and bDNA periods. We included patients with stable CD4 counts, excluding patients with viral loads ≥1,000 copies/ml or any significant changes in therapy. Out of 4500 clinic patients, 419 patients (1588 viral loads) were included. 39% of viral loads were reported as detectable by RT-PCR vs. 5% reported as detectable by bDNA. The mean coefficient of variation was higher before vs. after assay change. We found an odds' ratio of 16.7 for having a viral load >75 copies/ml during the RT-PCR vs. bDNA periods.

**Discussion:**

These data support previous reports, suggesting that bDNA may more reliably discriminate between viral suppression and low level viremia in stable patients on therapy. Low-level viremia, noted more with RT-PCR, may promote unneeded testing, while differences in viral load reliability may impact antiretroviral trial and quality assurance endpoints. Commonly used plasma separator tubes may differentially affect RT-PCR and bDNA results.

## Introduction

The accurate and reliable quantification of HIV-1 RNA levels, or plasma viral load (pVL), has become a crucial tool in the management of HIV disease. Providers use pVL to determine a patient's viral set point prior to the initiation of antiretroviral therapy (ART), to help decide when to initiate therapy, to monitor response to treatment and to detect treatment failure [1], [2]. For patients on therapy, and for their providers, viral load testing answers the vital question of whether ART has successfully suppressed their viremia. Assays used to quantify viral load should be able to help differentiate patients with adequate viral suppression (i.e. those who are undetectable) from patients with low level viremia, who may be failing therapy.

Two assays employed clinically to measure HIV-1 pVL currently predominate in the U.S., the reverse transcription polymerase chain reaction assay (RT-PCR) (AMPLICOR HIV-1 MONITOR Ultrasensitive version 1.5, Roche Molecular Systems, Inc) and the branched chain DNA assay (bDNA, VERSANT HIV-1 RNA version 3.0 bDNA Assay, Siemens Diagnostics). The current versions of these assays yield well correlated results throughout their dynamic ranges [3]–[5]. Yet, significant test performance differences exist, including differences in the assays' reproducibility near their lower limit of quantification (LLOQ) [6]–[8].

Peter and Blum report cumulative results for HIV-1 pVL testing done at a reference lab between January 2000 through December of 2001, with approximately 4000–7000 tests performed per month [8]. In September of 2000 their lab changed HIV-1 viral load assays, from RT-PCR (Roche AMPLICOR, version 1.5) to bDNA (Bayer VERSANT, version 3.0). They found the bDNA assay to be more reproducible at low copy numbers (75 copies/ml) than RT-PCR, with coefficients of variation (CV) of 20% versus 79% respectively [8].

Differing rates of reliability near the LLOQ between the RT-PCR and bDNA may have important ramifications for individual patient care. Despite the clinically innocuous nature of intermittent viremia under 200 copies/ml, these “blips” in otherwise stable patients may promote both patient and practitioner anxiety, leading to more frequent office visits, more laboratory testing, and possibly unneeded changes or intensification of anti-retroviral regimens [9]–[11]. Additionally, the presence of clinically significant differences in assay reliability raises the question of whether clinical trial data are comparable if different pVL assays are utilized. With the time to loss of virologic response (TLOVR) clinical trial end point advocated by the Food and Drug Administration (FDA) for new drug approval applications, failure is defined by two pVL measurements above the HIV RNA assay's LLOQ [12]. Trials which utilize RT-PCR may overestimate the rate of failure due to the assay's inherent variability near its LLOQ [13], [14].

The John H. Stroger, Jr. Hospital (JSH) of Cook County lab, which performs HIV-1 pVL testing for the Ruth M. Rothstein CORE Center, Cook County Health and Hospital System ambulatory HIV clinic, changed from using a RT-PCR assay to a bDNA assay in February 2005. During the entire period under review samples were collected in Plasma Preparation Tubes (PPTs). Despite the adverse effect that use of PPTs may have on RT-PCR reliability, they are widely used since they allow phlebotomy technicians to do a simple centrifugation step before forwarding specimens to the molecular diagnostics lab. Following our lab's change in methodology, our clinic quality assurance surveillance detected an increase in the proportion of patients with viral suppression (pVL<75 copies/ml) [11], [13].

We utilized a retrospective cohort study, selecting a group of immunologically stable patients, to determine if significant differences in the proportion of HIV pVLs reported as detectable existed between the RT-PCR versus bDNA assays.

## Methods

### Ethics statement

This research was approved by the Cook County Health and Hospital System's (CCHHS) Institutional Review Board. Since this research entailed the retrospective review of pre-existing data and because all personal identifying information has been permanently removed from the study database, the CCHHS IRB deemed that specific patient informed consent was not necessary.

We carried out a retrospective, two period review between May 2004 and August 2005, comparing HIV-1 pVL results for a cohort of highly stable patients. The JSH lab switched from the RT-PCR to the bDNA HIV pVL assay on February 1^st^, 2005. We conducted a review of the electronic medical record for the CORE Center's 4500+ patients. We included and compared all pVLs of patients who had ≥1 pVL assessed via RT-PCR (Roche AMPLICOR HIV-1 MONITOR Ultrasensitive version 1.5, Roche Molecular Systems, Inc) during eight months prior to assay change, and via bDNA (VERSANT HIV-1 RNA version 3.0 bDNA Assay, Siemens Diagnostics) during six months after the change. In order to exclude patients with recent viral decay, patients had to have at least one undetectable pVL during the six months prior to entering the review period. To select patients with viremia related to assay reliability differences, rather than medication non-adherence or overt virologic failure, we excluded patients with any pVL measurement ≥1,000 copies/ml during the eight month RT-PCR period or the six month bDNA period. Also, we excluded patients if they had a decrease in absolute CD4 count of ≥15% during the 14 month review period. We performed chart reviews, excluding patients with significant changes in ART either during the 14 month review period, or three months prior to entering the review period. Decisions regarding whether to exclude or include patients with changes in ART were made independent of knowledge of their pVL results.

To assess the assays' clinical reliability near their LLOQ, as the primary analysis, we compared the proportion of pVLs reported as detectable during each of the two periods. Test reliability may be defined as the extent to which test results remain consistent over repeat measurements of the same subject under similar conditions. A test is reliable if it yields consistent results, given stable testing conditions. It is judged not to be reliable if repeat measurements, under the same conditions, give different results. For patients in this stable cohort, all of whom demonstrated previous virologic suppression, continued on stable ART and maintained steady CD4 counts, we characterized test reliability by the proportion of samples with suppressed vs. detectable viremia during the RT-PCR vs. bDNA periods, with more episodes of detectable, low-level viremia signifying less clinical reliability near the assays' LLOQ. To determine an odd's ratio describing the likelihood for detectable viremia during the RT-PCR vs. bDNA periods, we utilized a conditional, fixed-effects, logistic regression model that accounted for correlation between an individual patient's multiple samples (Stata version 9, StataCorpLP, College Station, TX). To account for RT-PCR's slightly lower LLOQ (down to pVL of 50) vs. bDNA (LLOQ down of 75), for analysis purposes, RT-PCR results between 50 and 75 copies/ml were considered undetectable. .

As secondary endpoints, we compared censored mean pVLs and mean coefficients of variation (CV). In calculating censored means, we assign undetectable pVLs a value of 49 and 74 copies/ml for the RT-PCR and bDNA periods. As a sensitivity analysis we also calculated and report censored means using values of 49 copies/ml and 1 copy/ml for all undetectable pVLs, during both periods. Coefficients of variation (CV), representing the standard deviation divided by the mean, were calculated for each patient with more than one value per period. We report mean CVs using both censored values and actual values, after the exclusion of undetectable results. We do not report tests of statistical significance comparing censored mean pVLs, since the required left-sided censoring (i.e. undetectable  = 49, 74 or 1 copy/ml) is unlikely to reflect the true distribution of pVL values below the LLOQ. In order to assess utilization of pVL testing, we also compared mean duration between pVL measurements for the RT-PCR vs. bDNA periods.

## Results

Out of 4500+ clinic patients, 454 patients met initial inclusion criteria. Following chart review, a total of 419 patients (see Table 1 for clinical/demographic data) and their 1588 pVL measurements were included for analysis. We excluded 35 patients: 12 due to documented poor compliance with ART, 10 due to poor therapy history documentation, 9 related to their charts being at an inaccessible, off-site location, 2 who were lost to clinical follow-up but continued to have lab monitoring, and 2 who had ART discontinued due to medication side effects.

**Table 1:** Clinical/demographic characteristics and viral load distributions.

	N = 419
**Mean age (years)**	45
**Sex (% male)**	72%
**Race/ethnicity**	
** African-American**	53%
** Hispanic**	29%
** White**	15%
** Asian**	1%
** Other**	1%
** Unknown**	2%*
**Risk factor**	
** Hetero**	25%
** MSM**	24%
** Intravenous drug use (IDU)**	11%
** Peri-natal**	2%
** IDU/MSM**	1%
** Transfusion**	<1%
** Unknown**	37%
**Mean years with HIV**	7.5
**Antiretroviral regimen**	
**NRTI backbone +**	
** NNRTI**	54%
** PI**	33%
** NNRTI and PI**	6%
** NRTI a lone**	6%
** other**	1%
**Non-excluded change in ART**	44/419 (11%)
** ART change related to:**	
** Side effect/adverse effect of regimen**	35/44 (80%)
** Added Hepatitis B activity**	3/44 (7%)
** Poor CD4 response/intensification**	2/44 (5%)
** Decrease pill burden**	2/44 (5%)
** Pregnancy**	2/44 (5%*)
**Viral load Distributions**	
**RT-PCR period – number of viral loads**	836
** <50 copies/ml**	453/836 (54%)
** 50–75**	61/836 (7%)
** 76–200**	148/836 (18%)
** 201–400**	90/836 (11%)
** >400**	84/836 (10%)
**bDNA period – number of viral loads**	752
** <75 copies/ml**	717/752 (95%)
** 75–200**	24/752 (3%)
** 201–400**	6/752 (0.8%)
** >400**	5/752 (0.7%*)

* Rounding to whole numbers accounts for totals not equal to 100%.

**Figure 1:**
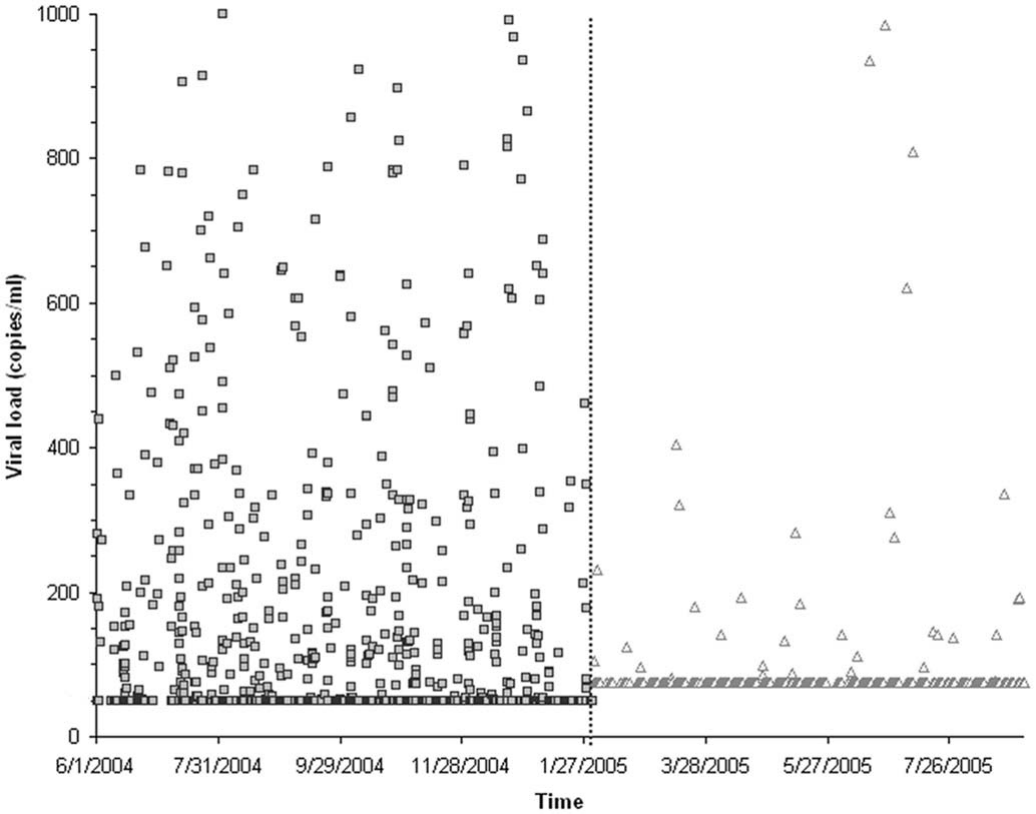
HIV-1 viral loads vs. time. This scatter plot shows each HIV viral load measurement (copies/ml on y-axis) vs. time (x-axis) with a vertical line dividing the RT-PCR (□ per each value) from bNDA (▵ per each value) time periods. In this figure, undetectable viral loads are censored to 49 copies/ml for the RT-PCR period and to 74 copies/ml for the bDNA period.

On average, each patient had 3.8 pVLs measured during the 14 month review period. During the RT-PCR period, 322/836 (39%) pVL values were ≥75 copies/ml vs. 35/752 (5%) during the bDNA period (*χ* ^2^ = 346, p<0.001) (see Table 1). Figure 1 illustrates pVL distributions for the two periods. By applying a conditional, fixed-effects, logistic regression model that matched each patient with him/herself throughout the observation period we sought to minimize patient introduced variation. We found an odds ratio of 16.7 (95% CI 10.7–26.1) for having a pVL≥75 copies/ml during the RT-PCR vs. bDNA periods.

The sensitivity analysis using different imputed values for undetectable viral loads demonstrates that using different values does not affect the finding that RT-PCR has a greater CV in these stable patients on invariant therapy who entered the observation period with undetectable pVL (see Table 2). After excluding the undetectable results, mean CVs were 0.55 (SD = 0.37) for the RT-PCR period vs. 0.19 (SD = 0.07) for the bDNA period (t = 5.69, p = 0.03), though only two patients had two detectable pVLs during the bDNA period from which a mean CV could be calculated. A per patient mean of 101 (SD = 31) vs. 104 (SD = 31) days elapsed between pVL measurements during the RT-PCR vs. bDNA periods (t = 1.10, p = 0.27).

**Table 2:** Mean censored viral loads and coefficients of variation.

	Undetectable to 49 copies/ml	Undetectable to 74 copies/ml	Undetectable to 1 copy/ml
Mean pVL RT-PCR period (SD), copies/ml	**149 (187)**	**Not calculated** *	**123 (203)**
Mean pVL bDNA period (SD), copies/ml	**57 (62)**	**81 (59)**	**12 (69)**
Mean CV RT-PCR period (SD)	**0.48 (0.45)**	**Not calculated** *	**0.76 (0.66)**
Mean CV bDNA period (SD)	**0.05 (0.19)**	**0.03 (0.14)**	**0.12 (0.41)**

* Value not calculated because RT-PCR LLOQ = 50 copies/ml, so for RT-PCR for values>50 actual, rather than censored values used in calculating means. We do not report tests of statistical significance comparing censored mean pVLs, since the required left-sided censoring (i.e. undetectable = 49, 74 or 1 copy/ml) is unlikely to reflect the true distribution of pVL values below the LLOQ.

## Discussion

These clinical data, drawn from a large group of immunologically stable, suppressed patients on established ART demonstrate that bDNA may more reliably discriminate between viral suppression and low level viremia in stable patients on therapy. Previous, similar reports comparing the assays' reliability lacked immunologic and treatment data to verify the clinical stability of patients with low-level viremia [6]–[8].

Several factors other than differences in assay reliability may have led to more detectable viremia during the RT-PCR period. RT-PCR has a lower reported LLOQ and 16% of the detectable pVLs during the RT-PCR period fell into the 50–75 copies/ml range. These values were considered undetectable for purposes of our primary analysis, thereby eliminating any difference mediated by this disparity in LLOQ. It should also be noted calculating censored mean pVL levels allowed for the reporting of mean CVs for the two periods, but it is unlikely that censoring of undetectable pVLs to an arbitrary, set value reflects the true distribution of pVLs below the LLOQ. Because of this, the secondary endpoint of difference in mean CV derived via use of the censored means should be cautiously interpreted.

Seasonal or time period bias may have contributed to differences noted in the two assays. Since we included over 400 patients on stable therapy and close to 1600 observations such effects are unlikely to have resulted in the magnitude of difference we demonstrated. Also, because loss of virologic control tends to increase with time, if anything, time period effects would have led to more detectable viremia during the chronologically later bDNA period.

The wide use of PPTs, rather than ethylenediaminetetraacetic acid (EDTA) tubes for collection of pVL specimens has been linked with false-positive, detectable values, especially when used with RT-PCR [11]. A study assessing for discordance between HIV pVL measured via RT-PCR, with specimens collected in ETDA-containing tubes versus PPTs, under varying processing protocols, demonstrated that transferring plasma from PPTs to a separate collection tube prior to specimen freezing eliminated the discordant, higher levels seen with specimens collected in PPTs vs. EDTA tubes [14]. These researchers also demonstrated that the bDNA assay, when compared to the RT-PCR assay, seemed less affected by the use of PPTs [15]. Giordano and colleagues evaluated Roche AMPLICOR MONITOR Ultra-sensitive versions 1.0 and 1.5 RT-PCR results from week 52 patients in a large, ART clinical trial. They found that 34% fewer patients would be categorized as virologic responders at week 52, as defined by having HIV-1 pVL<50 copies/ml, if PPTs vs. EDTA collection tubes were used [13].

Since we used PPTs during the entire RT-PCR and bDNA periods, collection tube effects likely influenced RT-PCR and bDNA assays differently, contributing to the observed differences in assay reliability near the LLOQ. Because PPTs require less specimen processing (e.g. no need to remove plasma from cellular components of sample), their use may improve lab technician safety and efficiency. Furthermore, clinicians may be unaware of these collection tube effects or of which collection tube their lab utilizes. The need to use EDTA tubes for RT-PCR rather than the more convenient PPTs may, therefore, present an additional barrier to effective HIV treatment monitoring. The data presented here demonstrate that bDNA presents a more clinically reliable option for quantifying HIV pVL versus RT-PCR in settings which use PPTs.

The difference we observed in clinical reliability at the lower end of the assays' dynamic ranges may have patient-care, quality assurance, and clinical research implications. While intermittent viremia of less than 200 copies/ml has been shown to be innocuous, sustained low-level viremia has been associated with the emergence of drug resistance [9], [16]. It is possible that the low-level viremia more frequently reported by RT-PCR may promote unneeded testing and/or medication changes [11]. While the mean time between pVLs measurements did not differ during the RT-PCR vs. bDNA periods at our institution, the difference in clinical reliability between the two assays may have led to differences in other, more difficult to measure outcomes, such as visit length and provider and/or patient anxiety.

Proportions of patients with undetectable viral loads are important benchmarks in clinical science and quality assurance. Since antiretroviral registrational trials utilize the TLOVR endpoint, RT-PCR's inferior reliability near the LLOQ, especially when using PPTs, may both inflate virologic failure rates and confound efforts to compare failure rates between trials [12]–[14]. Furthermore, comparing rates of HIV viral suppression between clinical care settings which use different assays should be approached with caution. Differences in the proportion of undetectable patients in clinics using bDNA vs. RT-PCR should be expected. Health insurers, state and federal payors, and federal funders who conduct benchmarking or quality assurance comparisons must be made aware of these differences when comparing clinical outcomes.

## References

[1] Mellors JW, Munoz A, Giorgi JV, Margolick JB, Tassoni CJ (1997). Plasma viral load and CD4 lymphocytes as prognostic markers of HIV-1 infection.. Ann Intern Med.

[2] Department of Health and Human Services (2006).

[3] Nolte FS, Boysza J, Thurmond C, Clark WS, Lennox JL (1998). Clinical comparison of enhanced-sensitivity branched-DNA assay and reverse transcription-PCR for quantitation of Human Immunodeficiency Virus type 1 RNA in plasma.. J Clin Micro.

[4] Elbeik T, Charlebois E, Nassos P, Kahn J, Hecht F (2000). Quantitative and cost comparison of ultrasensitive Human Immunodeficiency Virus type 1 RNA viral load assays: Bayer bDNA versions 3.0 and 2.0 and Roche PCR Amplicor Monitor version 1.5.. J Clin Micro.

[5] Galli R, Merrick L, Friesenhahn M, Ziermann (2005). Comprehensive comparison of the VERSANT HIV-1 RNA (bDNA) and COBAS AMPLICOR HIV-1 MONITOR 1.5 assays on 1000 clinical specimens.. J Clin Virol.

[6] Elbeik T, Alvord WG, Trichavaroj R, de Souza M, Dewar R (2002). Comparative analysis of HIV-1 viral load assays on subtype quantification: Bayer Versant HIV-1 RNA 3.0 versus Roche Amplicor HIV-1 Monitor version 1.5.. JAIDS.

[7] Murphy DG, Cote L, Fauvel M, Rene P, Vincelette (2000). Multicenter comparison of Roche COBAS Amplicor Monitor version 1.5, Organon Teknika NucliSense QT with Extractor, and Bayer Quantiplex version 3.0 for quantification of human immunodeficiency virus type 1 RNA in plasma.. J Clin Micro.

[8] Peter JB, Blum R (2002). Monitoring HIV viral loads in the United States: recent trends and methodologies.. JAIDS.

[9] Nettles RE, Kieffer TL, Kwon P, Monie D, Han Y (2005). Intermittent HIV-1 viremia (blips) and drug resistance in patients receiving HAART.. JAMA.

[10] Sungkanuparph S, Overton ET, Seyfried W, Groger R, Fraser V (2005). Intermittent episodes of detectable HIV viremia in patients receiving nonnucleoside reverse transcriptase inhibitor-based or protease inhibitor-based highly active antiretroviral therapy regimens are equivalent in incidence and prognosis.. Clin Infect Dis.

[11] Murphy R, Berzins B, Leake A, Till M, Stosor V (2005).

[12] Division of Antiviral Drug Products, Office of Drug Evaluation IV, Center for Drug Evaluation and Research (CDER), Food and Drug Administration. Guidance for Industry: Antiretroviral Drugs Using Plasma HIV RNA Measurements—Clinical Considerations for Accelerated and Traditional Approval. Accessed June 26, 2006. Available at: http://www.fda.gov/cder/guidance/3647fnl.pdf

[13] Giordano M, Kelleher T, Colonno RJ, Lazzarin A, Squires K (2006). The effects of the Roche AMPLICOR HIV-1 MONITOR UltraSensitive Test versions 1.0 and 1.5 viral load assays and plasma collection tube type on determination of response to antiretroviral therapy and inappropriateness of cross-study comparisons.. J Clin Virol.

[14] Ribaudo H, Lennox J, Currier J, Kuritzkes D, Gulick R (2009). Virologic failure endpoint definition in clinical trials: is using HIV-1 RNA threshold <200 copies/ml better than <50 copies/ml?.

[15] Salimnia H, Moore EC, Crane LR, MacArthur RD, Fairfax MR (2005). Discordance between viral loads determined by Roche COBAS AMPLICOR Human Immunodeficiency Virus type 1 Monitor (version 1.5) standard and ultrasensitive assays caused by freezing patient plasma in centrifuged Becton-Dickinson Vacutainer brand Plasma Preparation Tubes.. J Clin Micro.

[16] Nettles RE, Kieffer TL, Simmons RP, Cofrancesco J, Moore RD (2004). Genotypic resistance in HIV-1-infected patients with persistently detectable low-level viremia while receiving highly active antiretroviral therapy.. Clin Inf Dis.

